# Acute Hyperglycemia-Induced Injury in Myocardial Infarction

**DOI:** 10.3390/ijms25158504

**Published:** 2024-08-04

**Authors:** Martino Pepe, Francesco Addabbo, Annagrazia Cecere, Rocco Tritto, Gianluigi Napoli, Palma Luisa Nestola, Plinio Cirillo, Giuseppe Biondi-Zoccai, Salvatore Giordano, Marco Matteo Ciccone

**Affiliations:** 1 Division of Cardiology, Department of Interdisciplinary Medicine (D.I.M.), University of Bari “Aldo Moro”, 70100 Bari, Italymarcomatteo.ciccone@uniba.it (M.M.C.); 2 ASL Taranto, Local Health Authority of Taranto, Statistics and Epidemiology Unit, 74100 Taranto, Italy; francesco.addabbo@asl.taranto.it; 3 Division of Cardiology, Department of Cardiac, Thoracic and Vascular Sciences, University of Padua, 35128 Padua, Italy; annagrazia.cecere@unipd.it; 4 Division of Cardiology, Villa Verde Clinic, 74121 Taranto, Italy; gianluigi.napoli@libero.it; 5 Division of Cardiology, Mater Dei Hospital, 70125 Bari, Italy; luisanestola@libero.it; 6 Department of Advanced Biomedical Sciences, Federico II University of Naples, 80131 Naples, Italy; pcirillo@unina.it; 7 Department of Medical-Surgical Sciences and Biotechnologies, Sapienza University of Rome, 04100 Latina, Italy; gbiondizoccai@gmail.com; 8 Maria Cecilia Hospital, GVM Care & Research, 48032 Cotignola, Italy; 9 Division of Cardiology, Department of Medical and Surgical Sciences, “Magna Graecia” University, 88100 Catanzaro, Italy; sasigiordano@gmail.com

**Keywords:** diabetes mellitus, endothelium, coronary flow, inflammation, coagulation

## Abstract

Acute hyperglycemia is a transient increase in plasma glucose level (PGL) frequently observed in patients with ST-elevation myocardial infarction (STEMI). The aim of this review is to clarify the molecular mechanisms whereby acute hyperglycemia impacts coronary flow and myocardial perfusion in patients with acute myocardial infarction (AMI) and to discuss the consequent clinical and prognostic implications. We conducted a comprehensive literature review on the molecular causes of myocardial damage driven by acute hyperglycemia in the context of AMI. The negative impact of high PGL on admission recognizes a multifactorial etiology involving endothelial function, oxidative stress, production of leukocyte adhesion molecules, platelet aggregation, and activation of the coagulation cascade. The current evidence suggests that all these pathophysiological mechanisms compromise myocardial perfusion as a whole and not only in the culprit coronary artery. Acute hyperglycemia on admission, regardless of whether or not in the context of a diabetes mellitus history, could be, thus, identified as a predictor of worse myocardial reperfusion and poorer prognosis in patients with AMI. In order to reduce hyperglycemia-related complications, it seems rational to pursue in these patients an adequate and quick control of PGL, despite the best pharmacological treatment for acute hyperglycemia still remaining a matter of debate.

## 1. Introduction

Acute hyperglycemia is defined as a transient increase in plasma glucose levels (PGL) and is a common finding in both diabetic and non-diabetic patients admitted for acute myocardial infarction (AMI) [1,2]. As a matter of fact, although hyperglycemia has a higher prevalence in diabetics [3], only 20–25% of hyperglycemic AMI patients have a known history of diabetes [4,5]. 

Despite acute hyperglycemia being a frequent condition, no uniform and universally accepted cut-off values are available in the literature [6]. Therefore, its prevalence widely ranges from 20 to 50% of AMI patients, depending on the definition. In some cohorts of non-diabetic patients, an even wider variability has been reported with a prevalence of hyperglycemia ranging between 3% and 71% [7], even though about 31% of non-diabetic patients presenting with high PGL reveal afterward an undiagnosed diabetes [8]. Applying a cut-off value of >110 mg/dL, Kosiborod et al. found in a large cohort of about 140,000 elderly patients hospitalized for AMI that 50% suffered from acute hyperglycemia [4] (Table 1). Conversely, the American Heart Association Scientific Statement on Hyperglycemia and Acute Coronary Syndrome suggested an upper limit for admission “random glucose” level of 140 mg/dL since higher values were associated with higher 30-day and 1-year mortality [1,4]. The hypothesis that high PGL in the setting of AMI predicts short and long-term mortality, regardless of diabetes mellitus (DM) history, has been supported by several pieces of evidence over the last two decades [9,10,11,12,13,14,15,16]. Furthermore, hyperglycemia also appears to be associated with well-known negative prognostic predictors, such as anterior wall infarction, multivessel coronary artery disease (CAD), atrial fibrillation, more extensive myocardial necrosis, and worse left ventricular systolic function [17,18,19,20]. We aimed to provide a state-of-the-art literature review of the pathophysiological mechanisms through which hyperglycemia impacts patients’ prognosis, firstly impairing coronary artery flow.

## 2. Current Evidence: From the Bench to the Bedside

### 2.1. Pathophysiological Mechanisms Triggered by Hyperglycemic Status in the Setting of Acute Myocardial Infarction

Multiple pathophysiological mechanisms have been proposed to explain the adverse prognostic impact of acute hyperglycemia in AMI patients: platelet hyperactivity; upregulation of the coagulation cascade; induction of a pro-inflammatory state; and, above all, endothelial dysfunction [21].

### 2.2. Endothelial Dysfunction

Vascular endothelium cannot be considered a mere physical barrier between the circulating blood components and the underlying tissues; it plays a crucial regulatory role in the maintenance of vascular homeostasis [22,23]. Endothelial cells secrete mediators involved in the regulation of the vascular tone, platelet adhesion, leukocyte diapedesis, and coagulation/fibrinolysis balance. Consequently, when present, endothelial dysfunction causes the dysregulation of multiple critical pathways [24,25,26,27,28].

Over the last decades, several studies pointed out that prolonged hyperglycemia and acute fluctuations of PGL are responsible for impaired endothelial function in both macro- and microvascular circulation [29,30,31,32]. Back in 1998, Williams et al. proved that endothelium-dependent vasodilation was transiently decreased in healthy subjects facing hyperglycemic conditions [33]. Moreover, fluctuating plasma glucose levels, compared to persistent hyperglycemia, have been hypothesized to be more harmful in terms of microvascular and macrovascular complications as well as mortality [34]. Three metabolic pathways have been recently involved as the link between hyperglycemia and endothelial dysfunction: oxidative stress; dysregulation of the endothelial nitric oxide synthase (eNOS) activity; and overproduction of leukocyte adhesion molecules.

#### 2.2.1. Oxidative Stress

Increased oxidative stress can be considered the primary cause of endothelial dysfunction and is triggered by both PGL variability and prolonged high PGL [35,36,37]. Multiple mechanisms, mostly mutually interconnected, are accountable for the formation of reactive oxygen species (ROS): (1) enhanced activity of the mitochondrial respiratory chain (MRC); (2) activation of protein kinase C (PKC) isoforms; (3) increased polyol pathway flux associated with a reduction in endogenous antioxidant defenses; and (4) increased formation of advanced glycation end-products (AGEs).

**Figure 1:**
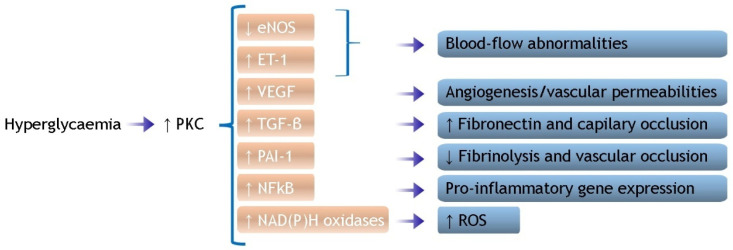
Effects of PKC activation induced by hyperglycemia. Activation of PKC inhibits eNOS expression and increases ET-1 activity, resulting in increased permeability of endothelium coupled with an improved expression of the permeability-enhancing factor VEGF in smooth muscle cells. PKC also contributes to increased microvascular matrix protein production through the expression of TGF-β and overexpression of the fibrinolytic inhibitor PAI-1. ENOS, endothelial nitric oxide synthetase; ET-1, endothelin-1; NAD(P)H, nicotinamide adenine dinucleotide phosphate; NFkB, nuclear factor kappa-light-chain-enhancer of activated B cells; PAI-1, plasminogen activator inhibitor-1; PKC, protein kinase C; TGF, transforming growth factor; VEGF, vascular endothelial growth factor.

- The MRC is a protein complex within the internal mitochondrial membrane, which is involved in the redox reactions aimed to produce adenosine triphosphate (ATP) by using oxygen as the final electrons’ acceptor. Some studies identify ROS overproduction by MRC as the principal cause of hyperglycemia-induced tissue damage through the increase in glycolysis, tricarboxylic acid (TCA) cycle activity, ATP/ADP ratio, and the hyperpolarization of the mitochondrial membrane [38,39,40,41]. When the overproduction of electron donors by the TCA cycle occurs, the increased electrochemical potential difference (generated by the proton gradient across the inner mitochondrial membrane) increases the production of superoxide anions by endothelial cells [42]. Moreover, hyperglycemia seems also responsible for the impairment of the mitochondrial ATP-sensitive potassium channels (mK_ATP_), which play a role in the protective preconditioning phenomenon [43,44];
- Several studies have also pointed out the PKC activation in vascular cells in response to high PGL [45,46,47]. PKC is triggered by intracellular signals, such as diacylglycerol (DAG) or calcium ions (Ca^2+^), whose concentration in endothelial cells increases during hyperglycemic states. PKC activation, in turn, via numerous different pathways (as described in Figure 1) contributes to the impairment of myocardial perfusion [42,48];
- In addition, a very suggestive discovery is that hyperglycemia stimulates the production of ROS in the heart through the activation of NADPH oxidase 2 (NOX2) via the sodiummyoinositol cotransporter-1 (SMIT1); the latter is an isoform of the sodium/glucose cotransporters (SGLT) and might explain some of the beneficial effects of the glifozines (SGLT-2 inhibitors) [49,50,51].

Moreover, hyperglycemia enhances the polyol pathway activity, resulting in the accumulation of fructose and sorbitol, which causes an imbalance in the intracellular redox state through an altered NADP+/NADPH ratio. The subsequent decrease in endogenous antioxidant defenses damages vascular homeostasis and intensifies the propensity to oxidative stress [42,46]. The increase in PGL also promotes the production of AGEs through non-enzymatic protein glycosylation and cross-linking reactions. These glycated proteins have been suggested to bind to specific receptors for AGEs (RAGE), which, in turn, activate NADP oxidase and eventually favor intracellular oxidative stress [52]. In detail, RAGEs, through a number of signaling cascades via phosphatidylinositol-3 kinase (PI3K), MAPK (ERK1 and 2), and Ki-Ras pathways, activate the nuclear factor-κB (NF-κB), favoring the production of ROS and the consequent cellular damage and mitochondrial dysfunction. NF-κB sub-unit p65 translocates into the nucleus to transcribe a number of pro-inflammatory cytokines and chemokines, among which are tumor necrosis factor α (TNF α), interleukin-6 (IL-6), monocyte chemoattractant protein-1 (MCP-1), vascular cell adhesion molecule-1 (VCAM-1), and intercellular adhesion molecule-1 (ICAM-1), which also promote inflammation and stimulate immune cells [53].

#### 2.2.2. Nitric Oxide Metabolism

**Figure 2:**
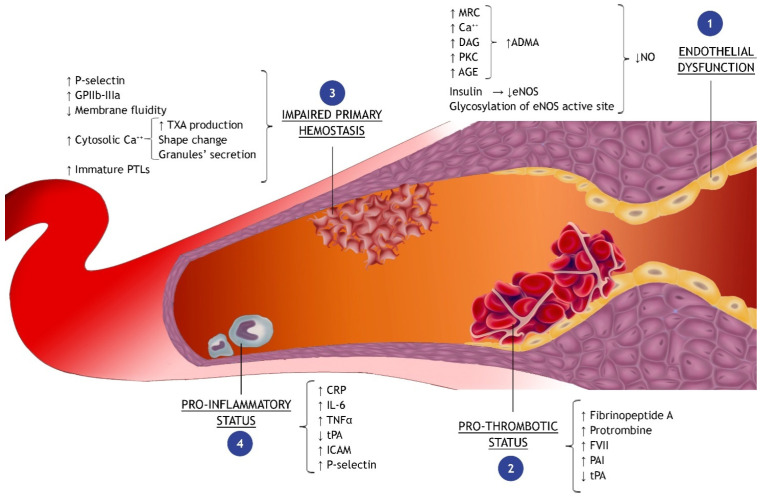
Mechanism of hyperglycemia-induced injury in myocardial infarction. ADMA, asymmetric dimethylarginine; AGE, advanced glycation end-products; CRP, C-reactive protein; DAG, diacylglycerol; eNOS, endothelial nitric oxide synthase; FVII, coagulation factor VII; GP, glycoprotein; ICAM, intercellular adhesion molecule; IL, interleukin; MRC, mitochondrial respiratory chain; NO, nitric oxide; PAI, plasminogen activator inhibitor; PKC, protein kinase C; PTL, platelet; TNF, tumor necrosis factor; tPA, tissue plasminogen activator; TXA, thromboxane-A.

Nitric oxide (NO) is a highly reactive gaseous compound with a very short half-life [54] whose production is catalyzed by the endothelial NO-synthase (eNOS) through the conversion of L-arginine to L-citrulline [55]. NO plays a crucial role in vascular function, being both a potent vasodilator, particularly effective in muscular arteries, and an inhibitor of platelets’ adhesion and aggregation, of leukocytes’ adhesion and migration, and of smooth muscle cell (SMC) proliferation. Under physiological conditions, shear stress is the principal eNOS trigger; nevertheless, the enzyme may also be activated by signaling molecules such as bradykinin, adenosine, vascular endothelial growth factor (in response to hypoxia), adiponectin, insulin, and serotonin [39,56,57]. Acute exposure of the vascular endothelium to elevated PGL results in the dysregulation of eNOS activity and reduced release of NO [30,33]. Several molecular mechanisms have been proposed: (1) the O-linked glycosylation of the eNOS active site [58,59]; (2) the inhibition of the insulin-stimulated expression of eNOS through the activation of the PKC pathway [60]; and (3) the AGEs–RAGEs interaction [61] (Figure 2(1)). Oxidative stress and NO metabolism appear tightly interconnected: the first may impair the enzymatic activity of dimethylarginine-dimethylaminohydrolase (DDAH) and increase asymmetric dimethylarginine (ADMA) levels, which, due to a structural similarity to L-arginine, acts as a competitive antagonist that binds to the catalytic site of eNOS resulting in inhibition of NO production. In particular, a novel model of “subclinical” NOS pharmacological inhibition with an ADMA analog demonstrated an enhanced generation of superoxide anion, which can possibly target the enzymes of the Krebs cycle, causing, in turn, the reduction in the mitochondrial mass and a switch to glycolytic metabolism in the face of normoxia, a first step toward endothelial dysfunction [35].

Seldom, eNOS can also generate superoxide instead of NO; this process, called eNOS uncoupling, is strongly dependent on the availability of eNOS substrate (arginine) and cofactor (tetrahydrobiopterin (BH4)) [62,63]. Hyperglycemia takes part in this vicious circle by determining an excessive production of intracellular ROS, leading to an increased formation of peroxynitrite, which, in turn, may react with the BH4 [64]. In the case of low BH4 concentration, eNOS becomes uncoupled and transfers electrons to molecular oxygen instead of L-arginine, thus producing superoxide rather than NO [35,62,65]. Heitzer and colleagues demonstrated that a diet supplement of BH4 in diabetic patients was able to improve endothelium-dependent vasodilation, thus elegantly proving that uncoupled eNOS plays a role in diabetic endothelial dysfunction [66].

### 2.3. Impaired Primary Hemostasis and Pro-Thrombotic Status

Elevated PGL drives adverse effects on ischemic myocardium, also favoring a systemic pro-thrombotic state. Different mechanisms have been identified [67], among which are the shortening of the fibrinogen half-life and the increased production of fibrinopeptide A, prothrombin fragments, factor VII, and other coagulation zymogens [1,68,69]. Furthermore, in acute hyperglycemic mouse models, lower tissue plasminogen activator (tPA) and higher plasminogen activator inhibitor (PAI) levels have been clearly demonstrated [70] (Figure 2(2)). Additionally, in non-diabetic subjects, a correlation between AGE formations during hyperglycemia and both PAI-1 and fibrinogen levels supports the interplay between different biochemical pathways triggered by the hyperglycemic status [71]. Besides the positive modulation of the coagulative cascade, hyperglycemia also impairs the fibrinolytic process by inducing structural changes in the fibrin molecules caused by the glycation reactions, with the consequent formation of denser clots, more resistant to fibrinolysis [67].

As concerns primary hemostasis, hyperglycemia modulates platelets’ reactivity, adhesion, and activation through several mechanisms (partly already mentioned) such as the upregulation of surface proteins (P-selectin and GP IIb/IIIa), the decreased membrane fluidity mediated by the glycation of membrane structures, the platelets swelling (an osmotic consequence of the abnormal activation of the polyol pathway), the upregulation of PKC intracellular signaling, and the overproduction of oxidant species [1,72,73] (Figure 2(3)). Moreover, the elevated concentration of cytosolic calcium, observed in platelets exposed to high glucose levels, may also play a role in all the phases of platelet activation, from shape change to granule secretion and thromboxane production [73].

Finally, hyperglycemia also seems to accelerate platelet turnover, as demonstrated by the presence of a higher number of immature reticulated platelets, which are larger, more sensitive, and less responsive to antiplatelet drugs [72,74]. In platelets, because of the absence of a nucleus, the normal lifespan of 7–10 days is largely determined by the mitochondria, which regulate the energy metabolism but also platelet activation and apoptosis [75].

### 2.4. Pro-Inflammatory Status

Several in vitro and in vivo studies have demonstrated a positive association between PGL and markers of vascular inflammation. Most evidence refers to elevated concentrations of C-reactive protein, interleukin-6, and tumor necrosis factor-alfa. Interestingly, the latter has been shown to induce cardiomyocytes’ apoptosis and correlate with larger infarct size in ischemic animal models [76,77,78]. Clinical evidence supporting these findings has been provided in a study on non-diabetic patients undergoing coronary artery bypass grafting (CABG): a significant reduction in inflammatory markers has been reported in the group undergoing a tight preoperative glycemic control with glucose–insulin–potassium (GIK) solution [79]. Moreover, during hyperglycemic states, an overproduction of leukocyte adhesion molecules [28], such as intercellular adhesion molecule-1 (ICAM-1), and chemokines, such as P-selectin, has also been described. Specifically, P-selectin is a key element in the rolling and diapedesis process, the first step of leukocyte activation. Once activated, the leukocytes, through the release of oxygen-derived free radicals, proteolytic enzymes, and other cytokines, induce a pro-inflammatory state, which, in turn, is capable of sustaining a vicious cycle that leads to further leukocyte activation and endothelial dysfunction [27,80] (Figure 2(4)). The role of leucocytes in the setting of hyperglycemia during acute myocardial ischemia has been confirmed in rats: Hokama et al. demonstrated that early after coronary reperfusion, higher levels of leukocytes become trapped in myocardial capillaries and venules of animals with diabetes than in controls [81].

### 2.5. Autophagy

Another very promising area of pathophysiologic research is autophagy, which is the ubiquitous cellular process of recycling damaged components [82]. In fact, interventions that leverage autophagy could counteract the detrimental effects of hyperglycemia on the cardiovascular system [83]. While data on this promising field of investigation are still preliminary, it is plausible that, for instance, metformin, SGLT-2 inhibitors, such as dapagliflozin and empagliflozin, and other agents with potent cardiometabolic effects may improve intracellular processes and protect from acute hyperglycemic stress, with effects that may be, at least in part, independent from PGL modulation [20,84,85]. This field of research seems very promising, as demonstrated by the plethora of ongoing clinical trials on the topic (https://clinicaltrials.gov/search?cond=Cardiovascular%20Diseases&intr=SGLT2%20inhibitors%2F(Dapagliflozin%20and%20Empagliflozin); accessed on 10 July 2024).

### 2.6. The Close Relationship between Acute Hyperglycemia, Coronary Flow and Myocardial Perfusion

**Figure 3:**
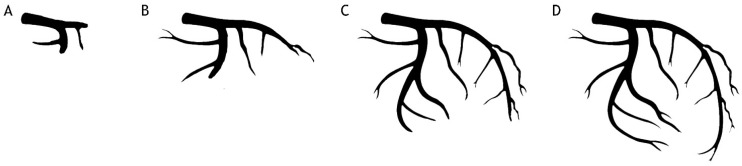
Elevated cTFC in patients with acute hyperglycemia (**A**). Coronary angiogram (CA) at the 25th cine frame (utilizing 30 fps acquisition), revealing contrast opacification only up to the proximal segment of LAD and CX. (**B**) CA at the 50th cine frame. (**C**) CA at the 75th cine frame. (**D**) CA at the 100th cine frame cTFC, showing full opacification of left coronary artery (normal value ≤ 25 frames).

Many epidemiological studies have found a high prevalence of acute hyperglycemia at presentation in patients with ST-segment elevation myocardial infarction (STEMI), regardless of DM history [86,87]. High PGL on admission is frequently associated with impaired coronary flow, which can be angiographically detected as “slow-flow” at presentation (TIMI flow grade 0–1) and/or “no-reflow”. The latter is defined as inadequate myocardial reperfusion after primary percutaneous coronary intervention (pPCI) despite effective treatment of the culprit lesion in the epicardial coronary artery with no angiographic evidence of epicardial obstruction, flow-limiting dissection, or vasospasm [12,88]. The impaired coronary flow has also been measured using the corrected TIMI frame count (cTFC): an easy, quantitative, and reproducible parameter reflecting both the epicardial flow and the microvascular function was used to more accurately assess the effectiveness of myocardial reperfusion after AMI [89]. Several pieces of evidence reported a higher cTFC in patients with acute hyperglycemia as proof of its negative impact on myocardial reperfusion and microvascular dysfunction [90] (Figure 3). This could be partially explained by the recruitment of activated leukocytes in coronary vessels, the subsequent pro-inflammatory status, and the impairment of myocardium oxygenation due to free radical overproduction, as previously described [81,91,92,93]. Moreover, the strict connection between hyperglycemia and no-reflow may also be due to additional mechanisms: first of all, a reduction in the collateral flow to the infarcted area through a NO-mediated mechanism, resulting in greater myocardial damage before reperfusion [94]; secondly, the hyperglycemia-induced platelet-dependent thrombosis [21], especially at capillaries level [95]; lastly, the detrimental impact of acute hyperglycemia on the cardioprotective mechanism of ischemic preconditioning (IPC), a powerful endogenous reaction against myocardial ischemia and reperfusion injury [96]. DM and acute hyperglycemia have been shown to counteract the positive effects of both ischemic and pharmacological preconditioning in animals and humans by inhibiting Akt phosphorylation. Noteworthy, the normalization of PGL determined by insulin administration has been conversely proved to be able to restore the cardioprotective effect of IPC [97].

Intriguingly, it was shown that both short- and long-term hyperglycemia increased the permeability and the barrier function of the endothelial glycocalyx and also decreased the functional capillary density and deformability of the red blood cells (RBCs). Glycocalyx also seems to have a putative role in myocardial tissue edema; thus, it could represent a potential early target of hyperglycemia [98].

Furthermore, in the course of AMI, the microvascular dysfunction in hyperglycemic patients is not limited to the culprit coronary artery since a 45% reduction in non-culprit coronary flow has also been demonstrated [99]. These data suggest a widespread dysfunction involving the whole coronary circulation rather than the thrombotic culprit lesion single-handedly. This hypothesis has been recently sustained in a selected cohort of STEMI patients divided into two groups based on PGL (< or >140 mg/dL): hyperglycemic patients showed higher cTFC values in both the culprit and non-culprit vessels regardless of preexisting diabetes. Moreover, a linear relationship between cTFC values and PGL at admission was also found [100]. The above-mentioned independence of the hyperglycemia-related microvascular damage from the possible concomitant presence of DM has been repeatedly demonstrated in the recent literature [101]. Planer et al. evaluated the prognostic significance of high PGL, regardless of DM diagnosis, in patients enrolled in the HORIZONS-AMI trial, a large-scale prospective study on STEMI patients. Patients with PGL > 200 mg/dL on admission showed a higher long-term mortality risk than the remaining population but comparable to subjects with a known history of DM [102]. Similar results had been previously reported by Stranders et al. [103]. Both studies concluded that high PGL at admission impacts short and long-term mortality independently of DM diagnosis, so hyperglycemia could be considered a supplementary risk factor. Conversely, in a recent analysis on 2958 consecutive STEMI patients, after stratification for DM history, hyperglycemia resulted as an independent predictor of mortality only in patients without DM [104]. The authors suggested, as a possible but speculative explanation of this finding, that chronic exposure to elevated glucose levels in diabetic patients could set the stage for a sort of protective “glucidic preconditioning” mechanism, which acts as a modulator of the detrimental effects of acute hyperglycemia during STEMI.

### 2.7. Glucose Control Strategy in Patients with AMI

The recognized correlation between acute hyperglycemia on admission and impaired vascular homeostasis suggests that insulin, due to its anti-inflammatory, anti-apoptotic, and pro-fibrinolytic properties, may be useful in the AMI setting to both achieve glycemic control and improve coronary flow [105,106]. Numerous preclinical studies supported the protective effect of early administration of insulin: possible mechanisms include the activation of eNOS and the eventual increase in NO synthesis [107,108], stimulation of angiogenesis, and inhibition of myocardial cell apoptosis [109]. Clinical data on insulin treatment are nevertheless non-univocal; it is known that chronic insulin treatment, within diabetic cohorts, is associated with worse prognosis in patients undergoing PCI because of either possible drug-related adverse effects (e.g., neointimal tissue proliferation, augmented plaque vulnerability, platelet dysfunction, resistance to antiplatelet agents, and vascular smooth muscle cell proliferation) or because insulin assumption may merely represent a marker of more advanced diabetic disease [39,110,111,112]. Evidence regarding the effectiveness of insulin use in the acute setting of AMI is also controversial: a sub-analysis of the DIGAMI study had shown that an intensive insulin regimen in diabetic patients with AMI [113] reduced long-term mortality compared to routine anti-diabetic therapy. Nevertheless, the attempt to confirm this finding failed with the following DIGAMI-2 study [114], which randomly assigned 1253 Type 2 DM (T2DM) patients with suspected acute coronary syndrome (ACS) to three different glycemic control strategies and showed no clinical benefit from an intensive insulin therapy. In the absence of conclusive data, the PGL management of these patients is still based on clinical common sense: before the publication of the 2023 European guidelines for the management of ACS [115] and Cardiovascular Disease in patients with Diabetes [116], the previous recommendation was to start a glucose-lowering therapy for glucose values above 180 mg/dL (class of recommendation IIa, level of evidence B) [117,118]. However, because of the lack of definite data, any cut-off value has been removed from the current guidelines and replaced with the broader definition of “persistent hyperglycemia” as an indication of pharmacological glycemic control [115,116]. According to this document, glycemic status should be assessed at initial evaluation in all patients with ACS and frequently monitored in patients with known diabetes or glucose levels exceeding 200 mg/dL.

## 3. Conclusions

Hyperglycemia on admission, regardless of DM history, has been identified as a strong predictor of impaired myocardial reperfusion and, thus, of worse outcomes in patients with AMI. High PGL should not be considered a mere stress-related epiphenomenon but an additional risk factor, which impacts short- and long-term prognosis through mechanisms involving endothelial dysfunction, platelet aggregation, inflammation, and oxidative stress [4,119,120]. In addition, in line with the above-described pathophysiology, high PGL at admission seems to impair the whole coronary circulation and not only the culprit vessel. To reduce hyperglycemia-related complications, it appears rational to pursue adequate glyco-metabolic control; anyhow, the best glycemic control strategy in the acute phase of AMI remains unknown and further studies are needed and strongly advocated.

## Figures and Tables

**Table 1:** Correlation between blood glucose level at presentation and 30-day/1 year mortality, according to Kosiborod et al. [4].

Blood Glucose Level at Presentation	% of Population	30-Days Mortality (%)	1 Year Mortality (%)
≤110 mg/dL	15.07	11	22.8
111–140 mg/dL	26.78	13.4	25.4
141–170 mg/dL	17.81	17.7	30.4
171–240 mg/dL	19.86	22.4	37.5
≥240 mg/dL	20.48	27.8	44.6
